# Treatment of Hay Fever

**Published:** 1902-08

**Authors:** 


### Treatment of Hay Fever.

MEANS of relief for this distressing trouble are many and varied but the evidence that adrenalin fully meets the indications as a remedial agent seems to be in the ascendency. It controls the nasal discharge, allays congestion of the mucous membrane, and in that manner reduces the swelling of the turbinal tissues. As the nasal obstruction disappears, natural breathing is materially aided and the ungovernable desire to sneeze is mitigated. Adrenalin blanches the mucous membrane by vigorously contracting the capillaries, and thus reduces local turgescence.

In the treatment of hay fever the solution of adrenalin chloride should be used. This preparation is supplied in the strength of one part adrenalin chloride to one thousand parts normal saline solution, and is preserved by the addition of 0.5 per cent. chloretone. The 1-1000 solution should be diluted by the addition of four parts normal salt solution and sprayed into the nares with a "cocaine" atomiser. In the office, the 1-1000 solution may be applied in full strength. A small pledget of cotton is wrapped about the end of an applicator and moistened with a few drops of the solution (1-1000), and the visible portions of the lower and middle turbinate bodies, and the septum, are carefully and thoroughly brushed. The same application is made to the other nostril, when usually relief follows in a few moments. Should the benefit prove only partial, the 1-5000 solution may now be sprayed into both nares, and a few drops instilled into both eyes. The effect of this treatment may be expected to last for several hours, one thorough application daily being sufficient to afford complete relief in many cases.

It is also recommended that solution adrenalin chloride be administered internally in 5 to 10 drop doses, beginning ten days to two weeks prior to the expected attack. In explanation of the beneficial effect of the drug when used in this manner, the suggestion has been made that hay fever is essentially a neurosis, characterised by a local vasomotor paraylsis, affecting the blood supply of the eyes, nose, face and pharynx, and occasionally of the laryngeal and bronchial mucous membranes. Adrenalin overcomes this condition, restores the normal balance in the local blood pressure, and thus aids in bringing about a cure.

